# Glomerular C4d Deposition and Persistent Proteinuria in IgA Nephropathy: A Longitudinal Cohort Study

**DOI:** 10.3390/jcm15218682

**Published:** 2026-11-06

**Authors:** Yasemin Aktaş Savrun, Taha Enes Çetin, Betül Öğüt, İpek Işık Gönül, Ömer Faruk Akçay, Özant Helvacı, Galip Güz

**Affiliations:** 1 Department of Internal Medicine, Antalya Training and Research Hospital, Antalya 07100, Türkiye; yasaktas@hotmail.com; 2 Department of Nephrology, Faculty of Medicine, Gazi University, Ankara 06560, Türkiye; omerfaruk_akcay@yahoo.com (Ö.F.A.); drozant@hotmail.com (Ö.H.); galipguz@hotmail.com (G.G.); 3 Department of Pathology, Faculty of Medicine, Gazi University, Ankara 06560, Türkiye; betulcimer@gmail.com (B.Ö.); dripek@gmail.com (İ.I.G.)

**Keywords:** IgA nephropathy, C4d, complement, mesangial C4d, glomerular capillary C4d, proteinuria, kidney failure

## Abstract

**Background:** Complement activation contributes to the pathogenesis of immunoglobulin A nephropathy (IgAN), and glomerular C4d deposition is a tissue marker of lectin and classical pathway activation. Whether C4d deposition and its intraglomerular localization are associated with longitudinal kidney outcomes independently of baseline disease severity remains uncertain. **Methods:** This retrospective single-center cohort included 77 adults with biopsy-proven primary IgAN. C4d was assessed immunohistochemically and considered positive when present in >25% of non-sclerotic glomeruli; positive biopsies were classified according to mesangial or glomerular capillary localization, and staining confined to sclerotic segments or Bowman’s capsule was not considered positive. eGFR and proteinuria measured from baseline to 48 months were analyzed using linear mixed-effects models, with adjustment for baseline proteinuria, Oxford S1, and corticosteroid treatment. Kidney failure, defined as sustained eGFR < 15 mL/min/1.73 m^2^, maintenance dialysis, or kidney transplantation, was analyzed using Kaplan–Meier and Cox regression analyses. **Results:** Glomerular C4d deposition was present in 32 patients (41.6%), with mesangial and glomerular capillary deposition in 15 (19.5%) and 17 (22.1%), respectively. Baseline proteinuria, S1 frequency, and serum C3 and C4 levels did not differ significantly according to C4d status. Mesangial C4d deposition was associated with S1 lesions (80.0% vs. 51.6%; *p* = 0.046). C4d-positive patients had persistently higher proteinuria during follow-up (*p* = 0.002), and this association remained after adjustment (geometric mean ratio 1.67, 95% CI 1.07–2.61; *p* = 0.026). The eGFR trajectory did not differ according to C4d status (*p* = 0.808). Kidney failure occurred in 11 patients (14.3%) and was not associated with C4d positivity after adjustment for baseline eGFR (HR 0.96, 95% CI 0.23–4.09). Neither localization was independently associated with longitudinal outcomes. **Conclusions:** In IgAN, glomerular C4d deposition was associated with persistently higher proteinuria after adjustment for baseline proteinuria, Oxford S1 status, and corticosteroid treatment, but was not significantly associated with longitudinal eGFR changes or kidney failure. The clinical relevance of C4d localization requires further evaluation in larger cohorts with longer follow-up.

## 1. Introduction

Immunoglobulin A nephropathy (IgAN) is one of the most common primary glomerular diseases worldwide and is characterized by a markedly heterogeneous clinical course [1]. While kidney function remains stable for many years in some patients, others experience progressive loss of kidney function and ultimately develop kidney failure [2]. In addition to clinical variables such as proteinuria, kidney function, and blood pressure, histopathological features incorporated into the Oxford MEST-C classification are used for prognostic assessment [3,4]. Nevertheless, substantial differences in outcomes are observed even among patients with similar clinical and histopathological profiles [5]. This limits the ability to predict individual disease progression and highlights the need for additional prognostic markers.

Complement activation plays an important role in the pathogenesis of IgAN, together with the inflammatory response triggered by glomerular immune-complex deposition [6]. Growing evidence supports the involvement of the alternative and lectin complement pathways in the development and progression of the disease [7]. C4d is a stable tissue marker generated through the cleavage of C4b during activation of the classical and lectin pathways and, owing to its covalent binding to tissue, provides a persistent footprint of complement activation [8]. The general absence of C1q deposition in IgAN suggests that C4d deposition may predominantly reflect activation of the lectin pathway [9]. Accordingly, C4d has attracted increasing attention as a potential histopathological biomarker in IgAN.

The involvement of complement in IgAN is closely related to the deposition of galactose-deficient IgA1-containing immune complexes within the mesangium [10]. These deposits can promote local complement activation, thereby contributing to mesangial inflammation and subsequent glomerular injury [11]. Several complement components and regulatory proteins have been identified in glomerular deposits, supporting the concept that complement activation occurs locally within the kidney [12]. The site of complement activation within the glomerulus may also be relevant. The mesangium is the primary site of IgA immune complex deposition, whereas extension of deposits to the glomerular capillary wall brings complement activation into direct contact with the endothelium and has been associated with endocapillary proliferation and more severe disease [13,14]. Accordingly, mesangial and glomerular capillary wall C4d deposition may reflect distinct patterns of complement-mediated injury. Tissue markers of complement activation, such as C4d, may therefore provide additional information regarding ongoing glomerular injury beyond conventional clinical and histopathological parameters.

Previous studies have associated glomerular or mesangial C4d positivity with higher levels of proteinuria, poorer kidney function, and more severe histopathological lesions; in some cohorts, C4d-positive patients have also exhibited less favorable long-term kidney outcomes [15,16]. However, considerable methodological heterogeneity exists across studies regarding the definition and assessment of C4d staining, and the independent prognostic value of C4d in IgAN remains incompletely defined [17]. Most studies have focused on mesangial C4d, and the significance of glomerular capillary wall C4d deposition has rarely been examined separately. In addition, many have relied on single follow-up measurements and have not fully accounted for differences in baseline disease severity. In this study, we aimed to evaluate the associations of glomerular C4d deposition and its mesangial or glomerular capillary localization with clinical and histopathological characteristics, and to assess their relationship with longitudinal proteinuria, kidney function, and kidney failure using repeated measurements and adjustment for baseline disease severity in patients with biopsy-proven IgAN.

## 2. Materials and Methods

### 2.1. Study Design and Population

This retrospective observational cohort study included adult patients with biopsy-proven IgAN who were followed at the Department of Nephrology, Gazi University Faculty of Medicine, between 2008 and 2021. Initially, 93 patients aged ≥18 years with histopathologically confirmed primary IgAN were identified. Patients with secondary IgA nephropathy, IgA vasculitis, or coexisting kidney disease on biopsy were not included. Of these, 16 were excluded because archived kidney biopsy specimens were unavailable for C4d assessment. The remaining 77 patients constituted the final study cohort. Baseline demographic and clinical characteristics of included and excluded patients were compared to assess potential selection bias (Supplementary Table S1).

Demographic, clinical, laboratory, treatment, and outcome data were retrospectively retrieved from the institutional medical records. Data regarding follow-up duration, administered treatments, and kidney replacement therapy (KRT), including kidney transplantation, were recorded. The study protocol was approved by the Clinical Research Ethics Committee of Gazi University on 9 June 2021 (Decision No. 620). The study was conducted using retrospectively collected clinical data and archived kidney biopsy specimens, without any additional study-related intervention, laboratory testing, or hospital visit.

### 2.2. Histopathological and Immunohistochemical Evaluation

Kidney biopsy specimens had been routinely evaluated at the time of diagnosis using hematoxylin and eosin, periodic acid–Schiff, Jones methenamine silver, Masson’s trichrome, and Congo red staining. Histopathological lesions were classified according to the Oxford MEST-C classification, including mesangial hypercellularity (M), endocapillary hypercellularity (E), segmental glomerulosclerosis (S), tubular atrophy/interstitial fibrosis (T), and cellular/fibrocellular crescents (C). Only biopsies containing at least eight glomeruli were considered adequate [4]. All histopathological and immunohistochemical evaluations were performed independently by two pathologists who were blinded to the patients’ clinical and outcome data. Discordant assessments were resolved by consensus.

For C4d assessment, archived formalin-fixed, paraffin-embedded kidney biopsy specimens were used. Sections of 3.5 μm thickness were obtained and subjected to deparaffinization and antigen retrieval. Immunohistochemical staining for C4d was performed automatically using a rabbit monoclonal C4d antibody (clone SP91, ab183311; Abcam, Cambridge, UK) and the ultraView DAB IHC Detection Kit on a BenchMark XT automated staining platform (Ventana Medical Systems, Inc., Tucson, AZ, USA). A kidney allograft biopsy with antibody-mediated rejection was used as a positive control, and omission of the primary antibody served as the negative control [18].

C4d staining was assessed in non-sclerotic glomeruli and scored as negative (0) or positive (1); faint, nonspecific background staining was disregarded. A biopsy was classified as C4d-positive when more than 25% of non-sclerotic glomeruli showed C4d deposition [18]. The localization of glomerular C4d deposition was assessed separately in the mesangium and glomerular capillary walls. The presence of simultaneous staining in both compartments was also evaluated. Staining confined to segmentally sclerotic areas or Bowman’s capsule was recorded separately but was not considered positive, because C4d deposition in sclerotic or necrotic lesions may reflect nonspecific entrapment rather than immune complex-mediated complement activation [14]. The distribution of staining across glomeruli (focal or diffuse) and within individual glomeruli (segmental or global) was also assessed.

### 2.3. Clinical and Laboratory Data

Clinical and laboratory variables recorded at the time of kidney biopsy included age, sex, serum creatinine, estimated glomerular filtration rate (eGFR), blood urea nitrogen, serum albumin, total serum protein, hemoglobin, serum C3, C4, and IgA levels, total cholesterol, low-density lipoprotein (LDL) cholesterol, triglycerides, systolic and diastolic blood pressure, 24-h urinary protein excretion, and the presence of microscopic or macroscopic hematuria. Mean arterial pressure (MAP) was calculated as diastolic blood pressure plus one-third of the pulse pressure. eGFR was calculated using the 2021 CKD-EPI creatinine equation [19].

Serum creatinine, eGFR, and 24-h urinary protein excretion were additionally recorded at 6, 12, 24, 36, and 48 months and at the last available follow-up. Treatment data, including renin–angiotensin–aldosterone system blockade, corticosteroid therapy, and other immunosuppressive treatments, were retrieved from medical records. All immunosuppressive treatments were initiated after kidney biopsy. Corticosteroid therapy was administered for persistent proteinuria despite optimized supportive care, an active urinary sediment, rising serum creatinine, or a combination of these findings [20,21]. Oral corticosteroids were initiated at 0.5–1 mg/kg/day (prednisolone or equivalent) and subsequently tapered. Patients with crescentic lesions received intravenous pulse methylprednisolone, followed by oral corticosteroids. Maintenance corticosteroid dosing was adjusted according to the course of eGFR and proteinuria during follow-up, and treatment duration was recorded.

Additional immunosuppressive agents, including cyclosporine, mycophenolate mofetil, cyclophosphamide, and azathioprine, were prescribed according to the treating physician’s clinical assessment and the patient’s response to treatment. The selection, initiation, and duration of these treatments were individualized rather than determined by a uniform protocol. Treatment was generally continued according to clinical response and tolerability and was discontinued or reassessed when eGFR declined below 30 mL/min/1.73 m^2^ or treatment-limiting adverse effects occurred. Owing to the retrospective study design and variability in follow-up intervals, precise treatment durations and dose adjustments for additional immunosuppressive agents could not be reliably reconstructed for all patients.

### 2.4. Kidney Outcomes

Kidney function and proteinuria were assessed at baseline, 6, 12, 24, 36, and 48 months, and the last available follow-up. Longitudinal changes in eGFR and proteinuria were evaluated using repeated measurements obtained at these predefined time points.

The clinical kidney endpoint was kidney failure, defined as a sustained eGFR < 15 mL/min/1.73 m^2^, confirmed by a repeat measurement at least 28 days later; initiation of maintenance dialysis (hemodialysis or peritoneal dialysis) continued for at least 28 days, or kidney transplantation [22,23]. For time-to-event analyses, follow-up was calculated from the date of kidney biopsy to the first kidney failure event, and patients who remained free of kidney failure were censored at the date of their last available follow-up. One patient underwent preemptive kidney transplantation without prior dialysis; however, this patient had already fulfilled the predefined eGFR criterion, confirmed by measurements at least 28 days apart, before transplantation. Accordingly, this patient was counted as a single kidney failure event at the time the eGFR criterion was fulfilled rather than at the date of transplantation. C4d assessment was performed on the diagnostic biopsy obtained at the start of follow-up.

### 2.5. Statistical Analysis

Statistical analyses were performed using IBM SPSS Statistics, version 29.0 (IBM Corp., Armonk, NY, USA). The distribution of continuous variables was assessed using histograms, Q–Q plots, and the Shapiro–Wilk test. Continuous variables with approximately normal distributions are presented as mean ± standard deviation (SD), whereas non-normally distributed variables are presented as median [25th–75th percentile]. Categorical variables are presented as number and percentage.

Comparisons between two independent groups were performed using the independent-samples *t*-test for approximately normally distributed continuous variables and the Mann–Whitney U test for non-normally distributed variables. Categorical variables were compared using Pearson’s chi-square test when expected cell counts were adequate. Fisher’s exact test was used for 2 × 2 tables with small expected cell counts, and the Fisher–Freeman–Halton exact test was used for larger contingency tables when the assumptions of the chi-square test were not satisfied.

Clinical and histopathological characteristics were compared according to glomerular C4d status and, separately, according to mesangial and glomerular capillary C4d deposition. Baseline characteristics of included and excluded patients were also compared (Supplementary Table S1).

Longitudinal changes in eGFR and proteinuria were analyzed using linear mixed-effects models. Proteinuria was natural log-transformed before analysis. Models included glomerular C4d status, categorical time (baseline and 6, 12, 24, 36, and 48 months), and the C4d × time interaction as fixed effects. Restricted maximum likelihood estimation, an unstructured covariance matrix for within-patient repeated measurements, and the Satterthwaite approximation for degrees of freedom were used. Last-follow-up values were not included in these models because follow-up duration varied between patients, and missing observations were not imputed. To account for differences in baseline disease severity and treatment, an adjusted model was fitted for proteinuria measured at 6–48 months, including baseline log-proteinuria, Oxford S1, and corticosteroid treatment (coded as a binary variable indicating treatment exposure during follow-up) as covariates. Adjusted results are reported as estimated marginal means and as geometric mean ratios, calculated by exponentiating the adjusted mean difference and its 95% confidence interval (CI). Separate adjusted models were fitted for mesangial and glomerular capillary C4d deposition, with adjustment for the baseline value of the respective outcome, Oxford S1, and corticosteroid treatment.

Kidney failure-free survival was estimated using the Kaplan–Meier method and compared using the log-rank test; survival curves were truncated at 72 months because of the small number of patients remaining at risk thereafter. Cox proportional hazards regression was used to estimate hazard ratios (HRs) and 95% CIs. The proportional hazards assumption was assessed using log-minus-log plots and was not violated. Given the limited number of kidney failure events (*n* = 11), multivariable Cox models were kept parsimonious. Three models were fitted: adjustment for baseline eGFR (Model 1), baseline eGFR and baseline proteinuria (Model 2), and baseline eGFR and Oxford S1 (Model 3). Models 2 and 3 were considered exploratory sensitivity analyses.

An exploratory analysis was additionally performed among patients without segmental glomerulosclerosis (S0) (Supplementary Table S2). All tests were two-sided, and a *p* value < 0.05 was considered statistically significant. Exploratory analyses were not adjusted for multiple comparisons.

## 3. Results

### 3.1. Study Population and Baseline Characteristics

A total of 93 patients with biopsy-proven IgA nephropathy were initially evaluated. Of these, 16 were excluded because archived biopsy material was unavailable for C4d assessment, leaving 77 patients in the final cohort. Glomerular C4d deposition was identified in 32 patients (41.6%), whereas 45 patients (58.4%) were classified as C4d-negative. The baseline characteristics of the study population are presented in Table 1. The mean age at diagnosis was 37.2 ± 10.4 years, and 50 patients (64.9%) were male. Median baseline eGFR was 72.0 mL/min/1.73 m^2^ (IQR, 44.5–104.5), and median 24-h urinary protein excretion was 2097 mg/day (IQR, 1290–3342).

Baseline demographic and clinical characteristics were generally comparable between the C4d-positive and C4d-negative groups. Median proteinuria was numerically higher in C4d-positive patients (2503 vs. 1672 mg/day; *p* = 0.086), as was the prevalence of segmental glomerulosclerosis (68.8% vs. 48.9%; *p* = 0.083). However, neither difference reached statistical significance. No significant between-group differences were observed in baseline eGFR, serum creatinine, mean arterial pressure, serum complement levels, serum IgA, or lipid parameters. Corticosteroids were administered to 59 patients (76.6%), with a median treatment duration of 6 months (IQR, 6–8). Other immunosuppressive therapies were used in 22 patients (28.6%), more frequently in the C4d-positive group than in the C4d-negative group (40.6% vs. 20.0%; *p* = 0.073), although this difference was not statistically significant. The frequencies of glomerular C3, C1q, IgM, and IgG deposition on immunofluorescence were also comparable between groups.

Comparison of the 77 included patients with the 16 excluded patients revealed no significant differences in age, sex, baseline kidney function, serum albumin, hemoglobin, mean arterial pressure, or proteinuria (Supplementary Table S1).

### 3.2. C4d Localization and Histopathological Characteristics

Among the 77 patients, mesangial C4d deposition was observed in 15 patients (19.5%), and glomerular capillary C4d deposition was identified in 17 patients (22.1%). No overlap between mesangial and glomerular capillary C4d deposition was observed in our cohort. An additional 14 patients exhibited C4d staining restricted to sclerotic glomerular areas (*n* = 12) or Bowman’s capsule (*n* = 2) and were classified as glomerular C4d-negative for the primary analyses. The clinical and histopathological characteristics according to mesangial and glomerular capillary C4d staining are presented in Table 2.

Patients with mesangial C4d positivity had a significantly higher prevalence of segmental glomerulosclerosis than those without mesangial staining (80.0% vs. 51.6%; *p* = 0.046). No significant differences were observed in the other Oxford MEST-C components or baseline clinical and laboratory parameters. In contrast, glomerular capillary C4d positivity was not significantly associated with any individual Oxford MEST-C lesion or baseline laboratory parameter.

### 3.3. Longitudinal Changes in Kidney Function

**Figure 1:**
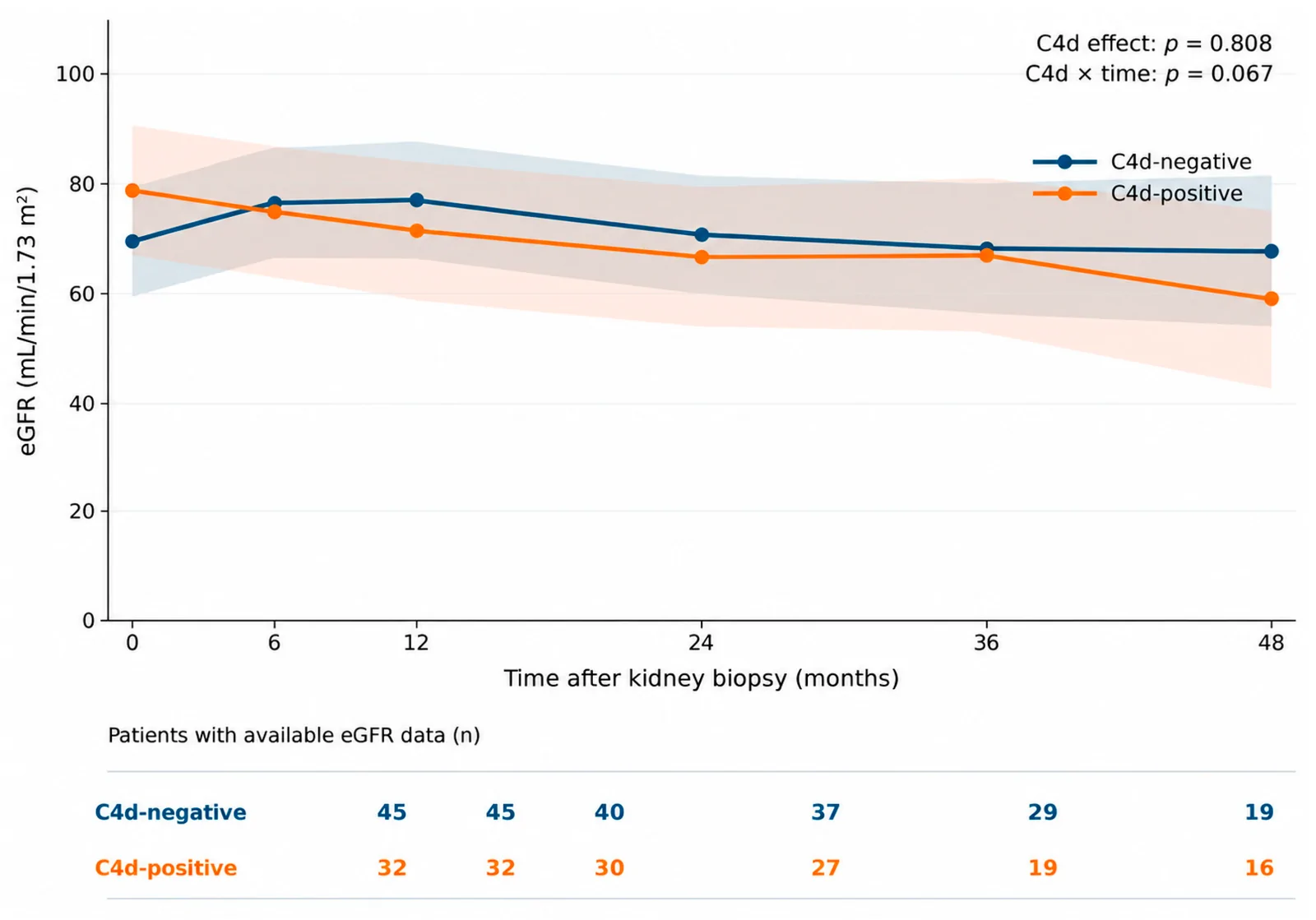
Longitudinal changes in estimated glomerular filtration rate according to glomerular C4d status. Estimated marginal means of eGFR from baseline to 48 months after kidney biopsy in C4d-negative (*n* = 45) and C4d-positive (*n* = 32) patients, derived from an unadjusted linear mixed-effects model including C4d status, categorical time, and the C4d × time interaction, with restricted maximum likelihood estimation and an unstructured covariance matrix. Shaded areas represent 95% confidence intervals. *p* values are shown for the effect of C4d status (*p* = 0.808), time (*p* < 0.001), and the C4d × time interaction (*p* = 0.067). The number of patients with available measurements at each time point is shown below the graph. eGFR was calculated using the 2021 CKD-EPI creatinine equation. eGFR, estimated glomerular filtration rate.

Longitudinal eGFR measurements were evaluated at baseline and at 6, 12, 24, 36, and 48 months following kidney biopsy. The numbers of patients with available measurements at each time point are shown in Figure 1.

At baseline, median eGFR was 54.0 mL/min/1.73 m^2^ in C4d-negative patients and 77.0 mL/min/1.73 m^2^ in C4d-positive patients (*p* = 0.224). At 48 months, the corresponding median values were 68.0 and 47.5 mL/min/1.73 m^2^, respectively (*p* = 0.282). None of the individual follow-up comparisons demonstrated a statistically significant difference between groups (Table 3). In the unadjusted linear mixed-effects model, eGFR changed significantly over time (*p* < 0.001), whereas neither the overall C4d effect (*p* = 0.808) nor the C4d-by-time interaction (*p* = 0.067) was significant (Figure 1).

### 3.4. Longitudinal Changes in Proteinuria

**Figure 2:**
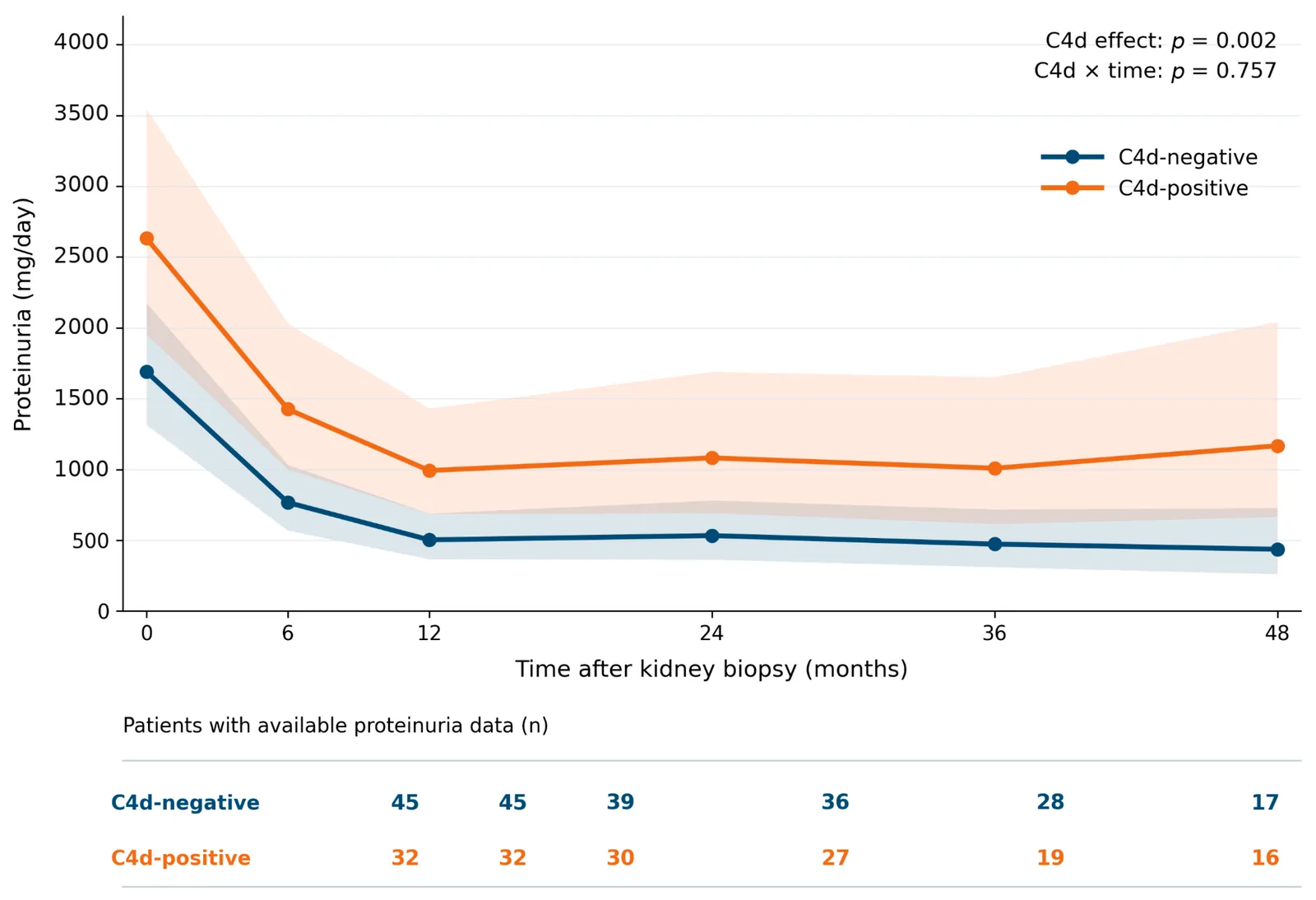
Longitudinal changes in proteinuria according to glomerular C4d status. Estimated marginal means of 24-h proteinuria from baseline to 48 months after kidney biopsy in C4d-negative (*n* = 45) and C4d-positive (*n* = 32) patients, derived from an unadjusted linear mixed-effects model of natural log-transformed proteinuria including C4d status, categorical time, and the C4d × time interaction, with restricted maximum likelihood estimation and an unstructured covariance matrix. Estimates and 95% confidence intervals (shaded areas) were back-transformed to the original scale (mg/day). *p* values are shown for the effect of C4d status (*p* = 0.002), time (*p* < 0.001), and the C4d × time interaction (*p* = 0.757). The number of patients with available measurements at each time point is shown below the graph.

Proteinuria decreased during follow-up in both groups, although C4d-positive patients generally exhibited higher proteinuria levels throughout the observation period (Table 3 and Figure 2).

At baseline, median 24-h proteinuria was 2503 mg/day in the C4d-positive group and 1672 mg/day in the C4d-negative group (*p* = 0.086). At 6 months, the corresponding values were 1270 and 869 mg/day (*p* = 0.015), and at 12 months, 1036 and 480 mg/day (*p* = 0.010). Higher proteinuria in the C4d-positive group was also observed at 24 months (*p* = 0.026) and 48 months (*p* = 0.011), but not 36 months (*p* = 0.069). At the last available follow-up, median proteinuria was 1316 mg/day in C4d-positive patients compared with 801 mg/day in C4d-negative patients (*p* = 0.005).

In the unadjusted longitudinal mixed-effects model using natural log-transformed proteinuria, a significant overall association with C4d status was observed (*p* = 0.002), together with a significant time effect (*p* < 0.001). However, the C4d-by-time interaction was not significant (*p* = 0.757), indicating that the difference between groups was not accompanied by statistically demonstrable differences in the temporal pattern of proteinuria change.

### 3.5. Adjusted Longitudinal Association Between C4d Deposition and Proteinuria

To evaluate whether the association between glomerular C4d deposition and proteinuria persisted after accounting for potential confounding factors, an adjusted longitudinal mixed-effects model was constructed using proteinuria measurements obtained at 6, 12, 24, 36, and 48 months. The model included glomerular C4d status, categorical follow-up time, their interaction, baseline log-transformed proteinuria, Oxford S1 status, and corticosteroid treatment (Table 4).

After adjustment, C4d positivity remained significantly associated with higher longitudinal proteinuria (*p* = 0.026). The adjusted mean difference in log-transformed proteinuria between C4d-positive and C4d-negative patients was 0.511 (95% CI, 0.063–0.958), corresponding to a geometric mean ratio of 1.67 (95% CI, 1.07–2.61). Thus, C4d-positive patients exhibited approximately 67% higher adjusted geometric mean proteinuria across follow-up assessments than C4d-negative patients.

Baseline log-proteinuria was also independently associated with longitudinal proteinuria (*p* = 0.001), while the overall time effect remained significant (*p* = 0.003). Oxford S1 status (*p* = 0.064) and corticosteroid treatment (*p* = 0.164) were not statistically significant. The adjusted C4d-by-time interaction was likewise not significant (*p* = 0.946).

### 3.6. Localization-Specific Longitudinal Associations

Separate adjusted longitudinal mixed-effects models were fitted to investigate the associations of mesangial and glomerular capillary C4d deposition with subsequent proteinuria and kidney function (Table 5). Mesangial C4d positivity was associated with a proteinuria ratio of 1.42 (95% CI, 0.81–2.49; *p* = 0.213), while glomerular capillary C4d positivity yielded a similar ratio of 1.42 (95% CI, 0.84–2.40; *p* = 0.186). Neither association reached statistical significance after adjustment.

For longitudinal eGFR, the adjusted mean difference was −3.21 mL/min/1.73 m^2^ (95% CI, −15.20 to 8.77; *p* = 0.594) for mesangial C4d positivity and −10.67 mL/min/1.73 m^2^ (95% CI, −21.44 to 0.11; *p* = 0.052) for glomerular capillary C4d positivity. No significant C4d-by-time interactions were identified in the localization-specific models for either proteinuria or eGFR.

### 3.7. Kidney Failure and Survival Analyses

**Figure 3:**
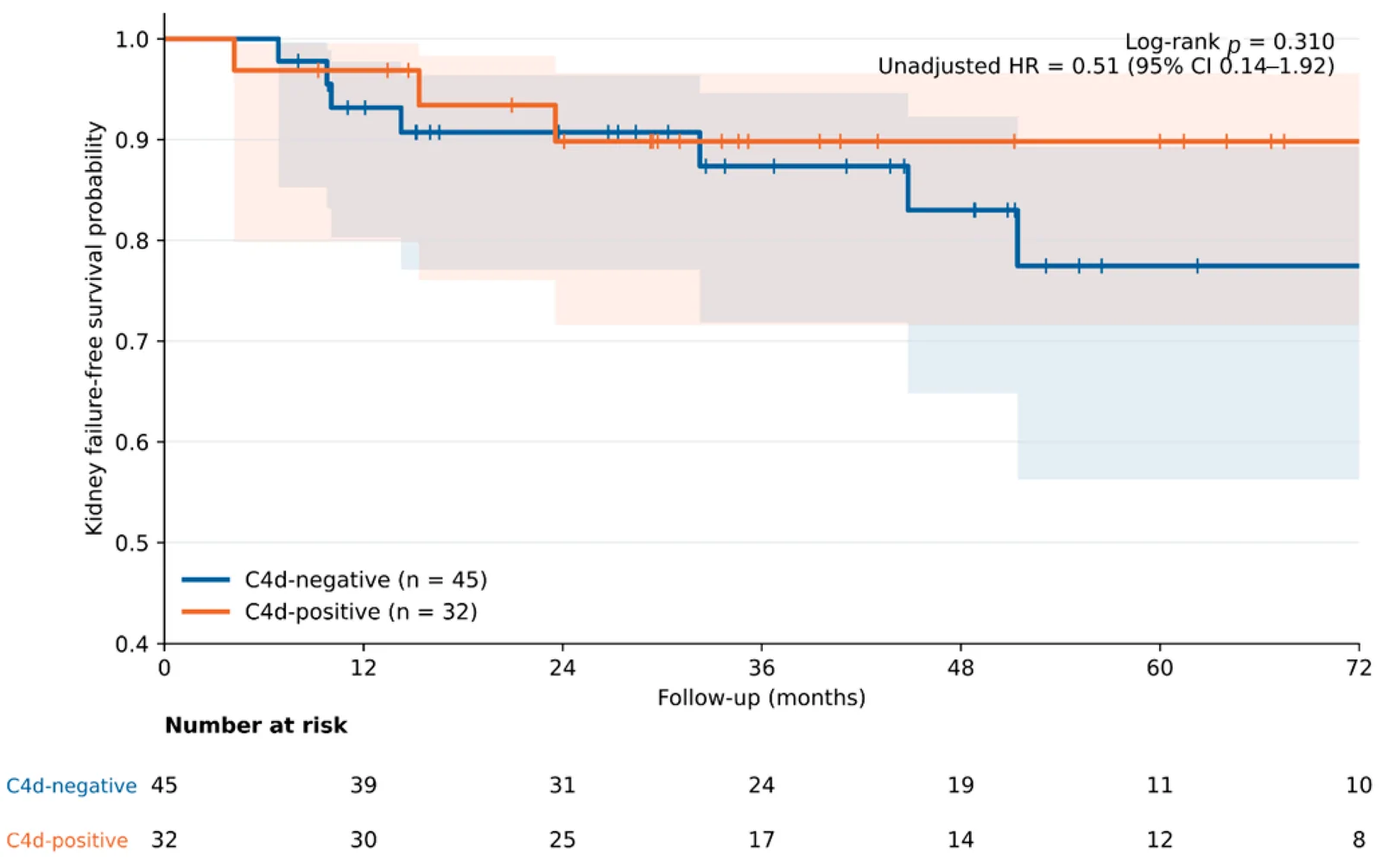
Kaplan–Meier curves of kidney failure-free survival according to glomerular C4d status. Kidney failure-free survival in C4d-negative (*n* = 45) and C4d-positive (*n* = 32) patients. Glomerular C4d positivity was defined as C4d deposition in >25% of non-sclerotic glomeruli, localized to the mesangium or glomerular capillary walls. Kidney failure was defined as a sustained eGFR < 15 mL/min/1.73 m^2^, initiation of maintenance dialysis, or kidney transplantation, and occurred in 8 (17.8%) C4d-negative and 3 (9.4%) C4d-positive patients. Survival distributions were compared using the log-rank test (*p* = 0.310); the hazard ratio was estimated using unadjusted Cox regression (HR 0.51, 95% CI 0.14–1.92). Shaded areas represent pointwise 95% confidence intervals, and vertical tick marks indicate censored observations. Numbers at risk are shown below the curves. Curves were truncated at 72 months because of the small number of patients remaining at risk thereafter. CI, confidence interval; eGFR, estimated glomerular filtration rate; HR, hazard ratio.

During follow-up, kidney failure occurred in 11 patients (14.3%), including 8 C4d-negative patients (17.8%) and 3 C4d-positive patients (9.4%). The difference in event frequency was not statistically significant (*p* = 0.345). Kaplan–Meier analysis showed no significant difference in kidney failure-free survival between the groups (log-rank *p* = 0.310). The unadjusted hazard ratio for kidney failure associated with C4d positivity was 0.51 (95% CI, 0.14–1.92), with a confidence interval encompassing substantial uncertainty (Figure 3).

Cox proportional hazards regression analyses are summarized in Table 6. In the unadjusted analysis, higher baseline eGFR was associated with a lower risk of kidney failure (HR per 10 mL/min/1.73 m^2^ increase, 0.622; 95% CI, 0.475–0.815; *p* = 0.001). After adjustment for baseline eGFR, glomerular C4d positivity was not significantly associated with kidney failure (adjusted HR, 0.959; 95% CI, 0.225–4.093; *p* = 0.955). The association remained nonsignificant in sensitivity models additionally accounting for baseline proteinuria or Oxford S1 status. Baseline proteinuria was associated with kidney failure in the model incorporating C4d status and baseline eGFR (adjusted HR per 1 g/day increase, 1.203; 95% CI, 1.043–1.387; *p* = 0.011). Similarly, Oxford S1 status was associated with increased kidney failure risk in the corresponding adjusted model (adjusted HR, 6.553; 95% CI, 1.200–35.793; *p* = 0.030). Given the limited number of kidney failure events, these adjusted Cox models were considered exploratory.

### 3.8. Subgroup Analysis in Patients Without Segmental Glomerulosclerosis

An exploratory subgroup analysis was performed among 33 patients without segmental glomerulosclerosis (Oxford S0), comprising 23 C4d-negative and 10 C4d-positive patients (Supplementary Table S2). At baseline, median eGFR was significantly higher in C4d-positive patients than in C4d-negative patients (113 vs. 73 mL/min/1.73 m^2^; *p* = 0.014). However, no significant differences in eGFR were observed at subsequent follow-up time points or at the last available assessment.

Baseline proteinuria was comparable between groups (1521.5 vs. 1672 mg/day; *p* = 0.906). At 12 months, C4d-positive patients had higher median proteinuria than C4d-negative patients (931.5 vs. 264 mg/day; *p* = 0.022). Differences at the remaining time points did not reach statistical significance. Kidney failure occurred in three C4d-negative patients and no C4d-positive patients (13.0% vs. 0%; *p* = 0.536).

## 4. Discussion

In this cohort of 77 patients with biopsy-proven IgAN, glomerular C4d deposition was present in 41.6% of biopsies. C4d positivity was associated with persistently higher proteinuria during four years of follow-up, and this association remained after adjustment for baseline proteinuria, Oxford S1, and corticosteroid treatment. In contrast, C4d positivity was not associated with the eGFR trajectory or with kidney failure. Mesangial C4d deposition was associated with S1 lesions, whereas neither localization was independently associated with longitudinal outcomes.

The prevalence of glomerular C4d positivity in our cohort lies within the range of approximately 22% to 55% reported previously [18,24]. This variability largely reflects differences in positivity thresholds, staining techniques, and the compartments evaluated. We applied an established threshold of >25% of non-sclerotic glomeruli and did not consider staining confined to sclerotic segments or Bowman’s capsule as positive, because C4d is nonspecifically deposited in sclerotic and necrotic lesions [14]. This distinction may be relevant: if staining in sclerotic areas is counted as positive, C4d positivity partly reflects the presence of segmental sclerosis itself. This may explain why the association between C4d and S1 in our cohort was weaker than that reported in some previous studies and did not reach statistical significance [17,25]. Notably, no overlap between mesangial and glomerular capillary C4d deposition was observed in our cohort. This observation may reflect the small sample size and should not be interpreted as evidence that the two patterns are biologically distinct or cannot coexist.

The main finding of our study is the persistent association between glomerular C4d deposition and proteinuria. Previous studies have reported higher proteinuria in C4d-positive patients, mostly at the time of biopsy [24,26]. In our cohort, baseline proteinuria did not differ significantly between the groups, whereas proteinuria remained higher in C4d-positive patients throughout follow-up despite a similar proportion receiving corticosteroids. The absence of a significant C4d × time interaction indicates that this difference was established early and persisted rather than widening over time. Importantly, the association remained after adjustment for baseline proteinuria, S1, and corticosteroid treatment, suggesting that C4d deposition is not merely a surrogate for more severe disease at presentation. Persistent proteinuria is among the strongest predictors of progression in IgAN, and proteinuria reduction is accepted as a surrogate endpoint in clinical trials [27,28,29]. C4d positivity may therefore identify patients in whom proteinuria responds less completely to supportive care and immunosuppression.

Several mechanisms may explain this association. In IgAN, C4d deposition in the absence of C1q is thought to reflect activation of the lectin pathway, and glomerular deposition of lectin pathway components has been associated with more severe histological damage and proteinuria [9,30]. Consistent with this, C1q deposition was rare in our cohort (5.2%) and did not differ according to C4d status. Serum C3 and C4 levels were within the normal range and similar between the groups, suggesting that C4d deposition primarily reflects local intraglomerular complement activation rather than systemic complement consumption [11]. Ongoing local complement activation may sustain mesangial and podocyte injury through the generation of anaphylatoxins and the membrane attack complex, thereby maintaining proteinuria even after the initial inflammatory activity has subsided [11,12].

Regarding localization, mesangial C4d deposition was associated with S1 lesions, a morphological feature of chronic glomerular injury that predicts progression in IgAN [31,32], whereas glomerular capillary C4d deposition showed no baseline clinicopathological associations. The mesangium is the primary site of IgA immune complex deposition, and mesangial complement activation may promote mesangial matrix expansion and segmental sclerosis over time. Capillary wall deposition, by contrast, places complement activation in direct contact with the endothelium and has been associated with endocapillary proliferation [13,14]; however, we did not observe an association with E1 lesions. Notably, neither localization was independently associated with longitudinal proteinuria or eGFR, although the point estimates for proteinuria were similar for both localizations. Given the small number of patients in each localization group, these analyses had limited power, and we cannot conclude that the two patterns have distinct clinical significance. A recent study in native kidney biopsies linked glomerular capillary C4d staining to a more proteinuric phenotype [33], and larger IgAN cohorts are needed to determine whether the localization of C4d deposition provides information beyond overall glomerular positivity.

C4d positivity was not associated with kidney failure in our cohort, in contrast to several previous studies [15,18,24,34,35]. Kidney failure was defined according to KDIGO criteria, including confirmed eGFR < 15 mL/min/1.73 m^2^, rather than KRT alone, to reduce the influence of factors unrelated to disease severity, such as transplantation opportunities [22]. The unadjusted hazard ratio favoured C4d-positive patients, but this estimate moved close to unity after adjustment for baseline eGFR, which was numerically lower in C4d-negative patients. This pattern indicates confounding by baseline kidney function rather than a protective effect of C4d. The small number of events (*n* = 11), the wide confidence intervals, and the relatively short median follow-up of approximately 42 months mean that a clinically relevant association cannot be excluded. Similarly, the C4d × time interaction for eGFR (*p* = 0.067) should not be interpreted as evidence of a difference in kidney function decline. Because sustained proteinuria precedes eGFR loss in IgAN, longer follow-up is needed to determine whether the proteinuria difference observed here translates into differences in kidney function.

The main methodological strength of our study is the longitudinal evaluation of proteinuria and kidney function using repeated measurements rather than relying solely on baseline and last-follow-up values. Furthermore, the association between glomerular C4d positivity and longitudinal proteinuria was evaluated after adjustment for baseline proteinuria, Oxford S1 status, and corticosteroid treatment. Standardized C4d staining criteria and blinded histopathological assessment supported the consistency of C4d classification.

Our findings should be interpreted in the context of several limitations. First, the retrospective, single-center design limits causal inference and generalizability. Archived biopsy material was unavailable for 16 patients, although their baseline characteristics did not differ from those of the included patients. Second, the localization-specific groups and the number of kidney failure events were small, limiting statistical power and allowing only parsimonious multivariable models; the exploratory analysis in S0 patients was also underpowered and is reported only in the Supplementary Materials. Third, treatment was not standardized and was guided by clinical indication, and practice may have changed over the 2008–2021 study period, so residual confounding related to treatment cannot be excluded. Fourth, the number of available measurements decreased during follow-up, and the mixed-effects models assume that missing data were missing at random. Finally, C4d was assessed only in glomeruli of the diagnostic biopsy by immunohistochemistry; arteriolar C4d, which has been associated with adverse outcomes in IgAN [36], lectin pathway components, and serial biopsies were not evaluated, so the proposed mechanisms could not be directly examined. In addition, serum complement assessment was limited to C3 and C4, as total hemolytic complement activity (CH50) and C1q levels were not routinely measured.

## 5. Conclusions

In patients with IgAN, glomerular C4d deposition was associated with persistently higher proteinuria during follow-up, independent of baseline proteinuria, segmental glomerulosclerosis, and corticosteroid treatment. C4d positivity was not associated with eGFR decline or kidney failure, and the clinical relevance of mesangial versus glomerular capillary localization remains uncertain. Larger prospective studies with longer follow-up are needed to determine whether glomerular C4d deposition identifies patients who may benefit from closer monitoring or complement-targeted therapies.

## Figures and Tables

**Table 1:** Baseline demographic, clinical, laboratory, histopathological, and treatment characteristics according to glomerular C4d staining status.

Characteristics	Overall (*n* = 77)	C4d-Negative (*n* = 45)	C4d-Positive (*n* = 32)	*p*-Value
Demographic characteristics				
Age at diagnosis, years	37.2 ± 10.4	38.19 ± 10.96	35.74 ± 9.59	0.312
Female sex, *n* (%)	27 (35.1)	16 (35.6)	11 (34.4)	0.915
Clinical characteristics				
Mean arterial pressure, mmHg	103.3 [93.3–110.0]	100.0 [93.3–107.5]	105.0 [93.3–119.2]	0.299
Macroscopic hematuria, *n* (%)	13 (16.9)	10 (22.2)	3 (9.4)	0.138
Microscopic hematuria, *n* (%)	57 (74.0)	34 (75.6)	23 (71.9)	0.717
Baseline laboratory parameters				
Serum creatinine, mg/dL	1.24 [0.91–1.77]	1.30 [0.91–1.87]	1.22 [0.87–1.58]	0.278
eGFR, mL/min/1.73 m^2^	72.0 [44.5–104.5]	54.0 [43.0–104.5]	77.0 [54.0–106.5]	0.224
Blood urea nitrogen, mg/dL	18.0 [14.4–26.1]	20.0 [13.9–28.0]	18.0 [14.2–24.2]	0.253
Serum albumin, g/dL	4.10 [3.70–4.40]	4.10 [3.70–4.40]	4.15 [3.63–4.38]	0.701
Hemoglobin, g/dL	13.68 ± 1.92	13.37 ± 1.92	14.13 ± 1.84	0.088
24-h urinary protein excretion, mg/day	2097 [1290–3342]	1672 [1180–3045]	2503 [1496–4354]	0.086
Serum C3, mg/dL	112.0 [104.3–128.0]	109 [104–119]	122 [103–140]	0.063
Serum C4, mg/dL	25.4 [21.9–28.9]	25.1 [20.3–28.4]	26.5 [23.9–30.0]	0.096
Serum IgA, mg/dL	260.0 [203.5–340.0]	266 [206–351]	249.5 [201.5–314]	0.319
Total serum protein, g/dL	6.79 ± 0.72	6.80 ± 0.61	6.76 ± 0.86	0.802
Total cholesterol, mg/dL	210.0 [179.0–248.5]	206 [179–247.5]	223 [182–256.5]	0.285
LDL cholesterol, mg/dL	130.0 [105.0–156.0]	126 [106.5–159]	138.5 [101.3–152]	0.691
Triglycerides, mg/dL	158.0 [118.5–219.5]	145 [106–201]	190.5 [138.5–232]	0.082
Oxford MEST-C classification				
Mesangial hypercellularity (M1), *n* (%)	47 (61.0)	29 (64.4)	18 (56.3)	0.467
Endocapillary hypercellularity (E1), *n* (%)	29 (37.7)	19 (42.2)	10 (31.3)	0.327
Segmental glomerulosclerosis (S1), *n* (%)	44 (57.1)	22 (48.9)	22 (68.8)	0.083
Tubular atrophy/interstitial fibrosis (T)				1.000 ᵃ
T0, *n* (%)	24 (31.2)	14 (31.1)	10 (31.3)	
T1, *n* (%)	44 (57.1)	26 (57.8)	18 (56.3)	
T2, *n* (%)	9 (11.7)	5 (11.1)	4 (12.5)	
Crescents (C)				0.921 ᵃ
C0, *n* (%)	56 (72.7)	32 (71.1)	24 (75.0)	
C1, *n* (%)	16 (20.8)	10 (22.2)	6 (18.8)	
C2, *n* (%)	5 (6.5)	3 (6.7)	2 (6.3)	
Immunofluorescence findings				
C3 deposition, *n* (%)	42 (54.5)	24 (53.3)	18 (56.3)	0.800
C1q deposition, *n* (%)	4 (5.2)	3 (6.7)	1 (3.1)	0.637 ᵇ
IgM deposition, *n* (%)	5 (6.5)	5 (11.1)	0 (0.0)	0.072 ᵇ
IgG deposition, *n* (%)	20 (26.0)	10 (22.2)	10 (31.3)	0.373
Treatment during follow-up (post-biopsy)				
Corticosteroid use, *n* (%)	59 (76.6)	32 (71.1)	27 (84.4)	0.175
Corticosteroid treatment duration, months	6 [6,7,8]	6 [6,7,8]	6 [6,7,8]	0.291
Other immunosuppressive therapy, *n* (%)	22 (28.6)	9 (20.0)	13 (40.6)	0.073 ᵇ
Cyclosporine, *n* (%)	11 (14.3)	5 (11.1)	6 (18.8)	—
Mycophenolate mofetil, *n* (%)	5 (6.5)	1 (2.2)	4 (12.5)	
Cyclophosphamide, *n* (%)	3 (3.9)	2 (4.4)	1 (3.1)	
Azathioprine, *n* (%)	3 (3.9)	1 (2.2)	2 (6.3)	

**Notes:** Overall glomerular C4d positivity was defined as C4d deposition in the mesangium and/or glomerular capillary walls. Staining confined to globally sclerotic glomerular areas or Bowman’s capsule was not considered positive; accordingly, 14 patients with these isolated staining patterns were included in the C4d-negative group. Data are presented as mean ± standard deviation, median [interquartile range (25th–75th percentiles)], or number (percentage), as appropriate. Between-group comparisons were performed using the independent-samples *t*-test, Mann–Whitney U test, Pearson’s chi-square test, or Fisher’s exact test, as appropriate. Superscript ᵃ indicates an exact test for multi-category comparisons; superscript ᵇ indicates Fisher’s exact test. Corticosteroid treatment duration was calculated only among patients who received corticosteroids (*n* = 59). Treatment variables refer to therapies administered after kidney biopsy. **Abbreviations:** eGFR, estimated glomerular filtration rate; LDL, low-density lipoprotein; MEST-C, mesangial hypercellularity (M), endocapillary hypercellularity (E), segmental glomerulosclerosis (S), tubular atrophy/interstitial fibrosis (T), and crescents (C).

**Table 2:** Baseline Clinical, Laboratory, and Histopathological Characteristics According to Mesangial and Glomerular Capillary C4d Staining Status.

Characteristics	Mesangial C4d− (*n* = 62)	Mesangial C4d+ (*n* = 15)	*p*-Value	Capillary C4d− (*n* = 60)	Capillary C4d+ (*n* = 17)	*p*-Value
Clinical characteristics						
Age at diagnosis, years	37.82 ± 10.77	34.49 ± 8.62	0.269	37.27 ± 10.48	36.85 ± 10.50	0.885
Female sex, *n* (%)	22 (35.5)	5 (33.3)	0.876	21 (35.0)	6 (35.3)	0.982
Mean arterial pressure, mmHg	101.7 (93.3–108.7)	106.7 (88.3–120.0)	0.718	100 (93.3–109.6)	103.3 (94.2–117.5)	0.375
Microscopic hematuria, *n* (%)	47 (75.8)	10 (66.7)	0.518 ᵃ	44 (73.3)	13 (76.5)	1.000 ᵃ
Macroscopic hematuria, *n* (%)	12 (19.4)	1 (6.7)	0.443 ᵃ	11 (18.3)	2 (11.8)	0.721 ᵃ
Laboratory characteristics						
Serum creatinine, mg/dL	1.24 (0.88–1.80)	1.35 (0.95–1.51)	0.837	1.31 (0.91–1.80)	1.21 (0.76–1.62)	0.274
eGFR, mL/min/1.73 m^2^	67 (44–105)	76 (54–102)	0.719	71 (43.3–103.5)	78 (50.5–113)	0.272
BUN, mg/dL	18 (13–27)	20 (15–24.3)	0.562	20 (15–26.1)	16 (11.5–23)	0.056
Serum albumin, g/dL	3.99 ± 0.50	4.08 ± 0.46	0.531	4.05 ± 0.47	3.86 ± 0.55	0.163
Hemoglobin, g/dL	13.60 ± 1.94	14.05 ± 1.81	0.415	13.54 ± 1.90	14.19 ± 1.92	0.215
24-h proteinuria, mg/day	2141 (1221–3321)	1829 (1495–5983)	0.625	1815 (1204–3064)	2760 (1465–4289)	0.116
Serum C3, mg/dL	110 (104.4–122)	128 (101–140)	0.153	112 (104.1–127.8)	119 (104–141.5)	0.400
Serum C4, mg/dL	25.35 (21–28.83)	26 (23–31)	0.319	25.2 (21.2–28.9)	27 (25–30)	0.305
Serum IgA, mg/dL	261 (204–342.5)	236 (184–320)	0.471	264 (201–349.3)	251 (203.5–309)	0.619
Total serum protein, g/dL	6.74 ± 0.66	6.98 ± 0.95	0.252	6.85 ± 0.71	6.57 ± 0.75	0.163
Total cholesterol, mg/dL	208 (179.5–244.8)	226 (166–259)	0.316	211 (178.5–251.3)	210 (187–241)	0.754
LDL cholesterol, mg/dL	128.5 (107.3–155.5)	138 (93–166)	0.995	128 (102.8–160)	139 (107.5–152)	0.641
Triglycerides, mg/dL	153 (117.3–208.5)	209 (146–302)	0.109	154 (112–221.8)	165 (131.5–215.5)	0.589
Oxford MEST-C classification						
M1, *n* (%)	37 (59.7)	10 (66.7)	0.618	39 (65.0)	8 (47.1)	0.181
E1, *n* (%)	24 (38.7)	5 (33.3)	0.700	24 (40.0)	5 (29.4)	0.426
S1, *n* (%)	32 (51.6)	12 (80.0)	0.046	34 (56.7)	10 (58.8)	0.874
T score			0.316 ᵇ			0.257 ᵇ
T0	18 (29.0)	6 (40.0)		20 (33.3)	4 (23.5)	
T1	35 (56.5)	9 (60.0)		35 (58.3)	9 (52.9)	
T2	9 (14.5)	0 (0.0)		5 (8.3)	4 (23.5)	
C score			0.695 ᵇ			0.515 ᵇ
C0	44 (71.0)	12 (80.0)		44 (73.3)	12 (70.6)	
C1	13 (21.0)	3 (20.0)		13 (21.7)	3 (17.6)	
C2	5 (8.1)	0 (0.0)		3 (5.0)	2 (11.8)	

**Notes:** Data are presented as mean ± standard deviation, median [interquartile range (Q1–Q3)], or number (percentage), as appropriate. Continuous variables were compared using Student’s *t*-test or the Mann–Whitney U test, and categorical variables using Pearson’s chi-square test or an appropriate exact test. Superscripts ᵃ and ᵇ indicate Fisher’s exact test and the Fisher–Freeman–Halton exact test, respectively. Mesangial and glomerular capillary C4d deposition were assessed separately, with no overlapping positivity observed. Localization-specific C4d-negative groups included patients positive in the other compartment; thus, their sample sizes (*n* = 62 and *n* = 60, respectively) differ from the overall C4d-negative group (*n* = 45) in Table 1. Patients with staining confined to sclerotic areas or Bowman’s capsule (*n* = 14) were considered negative in both comparisons. These analyses were exploratory, without adjustment for multiple comparisons. **Abbreviations:** C4d, complement component 4d; eGFR, estimated glomerular filtration rate; BUN, blood urea nitrogen; IgA, immunoglobulin A; LDL, low-density lipoprotein; MEST-C, Oxford Classification of IgA nephropathy (M, mesangial hypercellularity; E, endocapillary hypercellularity; S, segmental glomerulosclerosis; T, tubular atrophy/interstitial fibrosis; C, crescents).

**Table 3:** Longitudinal Renal Function, Proteinuria, and Kidney Outcomes According to Glomerular C4d Staining Status.

Parameter	C4d-Negative (*n* = 45)	C4d-Positive (*n* = 32)	*p*-Value
eGFR (mL/min/1.73 m^2^)			
Baseline	54.0 (43.0–104.5)	77.0 (54.0–106.5)	0.224
6 months	76.0 (52.0–104.0)	72.0 (45.3–99.3)	0.820
12 months	84.5 (54.3–111.8)	72.0 (48.8–96.8)	0.296
24 months	76.0 (42.5–99.5)	65.0 (46.0–91.0)	0.744
36 months	72.0 (48.0–98.0)	57.0 (45.0–91.0)	0.429
48 months	68.0 (34.0–87.0)	47.5 (44.0–67.8)	0.282
Last follow-up	72.0 (36.5–105.0)	53.5 (26.0–89.5)	0.230
Proteinuria (mg/day)			
Baseline	1672 (1180–3045)	2503 (1496–4354)	0.086
6 months	869 (347–1549)	1270 (908–2014)	0.015
12 months	480 (205–1149)	1036 (570–1697)	0.010
24 months	449 (153–1248)	1025 (520–1783)	0.026
36 months	357 (190–1212)	960 (488–1505)	0.069
48 months	301 (109–763)	1081 (386–1577)	0.011
Last follow-up	801 (255–1444)	1316 (758–2956)	0.005
Clinical outcomes			
Follow-up duration (months)	43.73 (20.12–67.68)	40.12 (25.38–72.09)	0.598
Kidney failure, *n* (%)	8 (17.8)	3 (9.4)	0.345
Longitudinal mixed-effects model results			
Outcome	C4d effect (*p*)	Time effect (*p*)	C4d × time (*p*)
eGFR	0.808	<0.001	0.067
Log-transformed proteinuria	0.002	<0.001	0.757

**Notes:** Data are presented as median (interquartile range, 25th–75th percentiles) or number (percentage), as appropriate. Between-group comparisons were performed using the Mann–Whitney U test or Fisher’s exact test, as appropriate. Longitudinal eGFR and natural log-transformed proteinuria were analyzed using unadjusted linear mixed-effects models with C4d status, categorical time, and their interaction as fixed effects. Models used restricted maximum likelihood estimation, an unstructured covariance matrix, and Satterthwaite’s approximation for degrees of freedom. Measurements at baseline and 6, 12, 24, 36, and 48 months were included; last-follow-up values were excluded because of variable follow-up durations. Missing observations were not imputed. **Abbreviations:** C4d, complement component 4d; eGFR, estimated glomerular filtration rate.

**Table 4:** Adjusted Association of Glomerular C4d Staining with Longitudinal Proteinuria Outcomes.

Parameter	Adjusted Estimate (95% CI)	*p*-Value
Adjusted proteinuria according to C4d status		
C4d-negative, mean log-proteinuria	6.393 (6.085–6.701)	
C4d-positive, mean log-proteinuria	6.904 (6.535–7.272)	
Adjusted difference (C4d-positive vs. negative)	0.511 (0.063–0.958)	0.026
Geometric mean ratio (C4d-positive vs. negative)	1.67 (1.07–2.61)	0.026
Longitudinal mixed-effects model	F statistic	*p*-value
Glomerular C4d status	5.207	0.026
Time	4.656	0.003
Oxford S1	3.549	0.064
Corticosteroid treatment	1.982	0.164
Baseline log-proteinuria	13.263	0.001
C4d × time interaction	0.183	0.946

**Notes:** Values are presented as adjusted estimated marginal means, mean differences, geometric mean ratios (all with 95% confidence intervals), or F statistics, as appropriate. Longitudinal log-transformed proteinuria (6–48 months) was analyzed using a linear mixed-effects model adjusted for baseline log-proteinuria, Oxford S1, corticosteroid treatment, categorical time, and the C4d × time interaction. The model used restricted maximum likelihood estimation, an unstructured covariance matrix, and Satterthwaite’s approximation. Estimated marginal means were averaged across follow-up time points, with baseline log-proteinuria held at its mean. Geometric mean ratios were obtained by exponentiating adjusted log-scale differences and their confidence limits. **Abbreviations:** C4d, complement component 4d; CI, confidence interval.

**Table 5:** Adjusted Longitudinal Associations of C4d Localization With Proteinuria and eGFR.

Outcome	C4d Localization	Adjusted Effect (95% CI)	*p*-Value	C4d × Time *p*-Value
Proteinuria	Mesangial C4d	1.42 (0.81–2.49)	0.213	0.815
	Glomerular capillary C4d	1.42 (0.84–2.40)	0.186	0.996
eGFR	Mesangial C4d	−3.21 (−15.20 to 8.77)	0.594	0.742
	Glomerular capillary C4d	−10.67 (−21.44 to 0.11)	0.052	0.322

**Notes:** Data are presented as adjusted effect estimates with 95% confidence intervals (CIs). Mesangial and glomerular capillary C4d deposition were evaluated in separate linear mixed-effects models, including categorical time, C4d status, and their interaction, adjusted for the corresponding baseline outcome, Oxford S1 status, and corticosteroid treatment. Follow-up measurements at 6, 12, 24, 36, and 48 months were included. Models used restricted maximum likelihood estimation, an unstructured covariance matrix, and Satterthwaite’s approximation for degrees of freedom. Proteinuria estimates are expressed as exponentiated ratios, whereas eGFR estimates represent adjusted mean differences (mL/min/1.73 m^2^), averaged across follow-up visits. For each localization, the reference group comprised patients without C4d deposition in that compartment, including those positive in the other compartment. **Abbreviations:** C4d, complement component 4d; CI, confidence interval; eGFR, estimated glomerular filtration rate.

**Table 6:** Cox Regression Analyses for Kidney Failure According to C4d Positivity and Baseline Clinical Characteristics.

Variables	Unadjusted HR (95% CI)	*p*-Value	Model 1 aHR (95% CI)	*p*-Value	Model 2 aHR (95% CI)	*p*-Value	Model 3 aHR (95% CI)	*p*-Value
C4d positivity	0.509 (0.135–1.922)	0.319	0.959 (0.225–4.093)	0.955	0.740 (0.135–4.050)	0.729	0.588 (0.135–2.569)	0.480
Baseline eGFR (per 10 mL/min/1.73 m^2^ increase)	0.622 (0.475–0.815)	0.001	0.624 (0.465–0.838)	0.002	0.569 (0.397–0.815)	0.002	0.527 (0.353–0.789)	0.002
Baseline proteinuria (per 1 g/day increase)	1.107 (0.992–1.236)	0.069	—	—	1.203 (1.043–1.387)	0.011	—	—
Oxford S1 (vs. S0)	2.144 (0.567–8.102)	0.261	—	—	—	—	6.553 (1.200–35.793)	0.030

**Notes:** Cox proportional hazards regression was used to evaluate associations with kidney failure. Model 1 included C4d positivity and baseline eGFR; Model 2 additionally included baseline proteinuria, whereas Model 3 included Oxford S1 instead of proteinuria. All models included 77 patients and 11 kidney failure events. C4d positivity was defined as mesangial and/or glomerular capillary deposition. Given the limited number of events, Models 2 and 3 were considered exploratory, and their estimates should be interpreted cautiously. **Abbreviations:** C4d, complement component 4d; HR, hazard ratio; aHR, adjusted hazard ratio; CI, confidence interval; eGFR, estimated glomerular filtration rate.

## Data Availability

The data supporting the findings of this study are available from the corresponding author upon reasonable request, subject to ethics approval and applicable data protection requirements.

## Supplementary Materials

The following supporting information can be downloaded at: https://www.mdpi.com/article/10.3390/jcm15218682/s1, Table S1: Comparison of baseline characteristics between included and excluded patients; Table S2: Kidney Function, Proteinuria, and Kidney Failure According to C4d Status in Patients Without Segmental Glomerulosclerosis (Oxford S0).
