# Supplementary material for: Glomerular C4d Deposition and Persistent Proteinuria in IgA Nephropathy: A Longitudinal Cohort Study
Source: J Clin Med. 2026 Nov 6;15(21):8682. doi: 10.3390/jcm15218682 (PMC13763832; doi:10.3390/jcm15218682)
Supplement: Supplementary file 1 [file jcm-15-08682-s001.zip › jcm-4611410-supplementary.pdf]

Supplementary Table S1. Comparison of baseline characteristics between included and excluded patients

**Characteristic**	**Included patients (n = 77)**	**Excluded patients (n = 16)**	**p-value**
Age, years	36.56 (28.94–45.29)	37.01 (29.54–43.24)	0.903
Female sex, n (%)	27 (35.1)	7 (43.8)	0.512
Serum creatinine, mg/dL	1.24 (0.91–1.77)	1.19 (0.71–1.65)	0.404
eGFR, mL/min/1.73 m^2	72.00 (44.50–104.50)	81.00 (45.50–109.00)	0.518
BUN, mg/dL	18.00 (14.40–26.05)	17.00 (12.75–25.75)	0.541
Serum albumin, g/dL	4.01 ± 0.49	4.12 ± 0.41	0.411
Hemoglobin, g/dL	13.68 ± 1.92	13.99 ± 2.24	0.576
Mean arterial pressure, mmHg	103.30 (93.30–110.00)	105.85 (96.70–120.45)	0.363
Proteinuria, mg/day	2097 (1290–3342)	2214 (1277–3002)	0.843

Notes: *Data are presented as mean ± standard deviation, median [interquartile range (25th–75th percentiles)], or number (percentage), as appropriate. Between-group comparisons were performed using the independent-samples t-test, Mann–Whitney U test, or Pearson's chi-square test, as appropriate. No statistically significant differences in the evaluated baseline characteristics were observed between included and excluded patients.* ***Abbreviations:*** *eGFR, estimated glomerular filtration rate; BUN, blood urea nitrogen.*

Supplementary Table S2. Kidney Function, Proteinuria, and Kidney Failure According to C4d Status in Patients Without Segmental Glomerulosclerosis (Oxford S0)

Variable	C4d-negative (n=23)	C4d-positive (n=10)	p-value
eGFR (mL/min/1.73 m²)			
Baseline	73 (43–104)	113 (61.5–128.3)	0.014
Month 6	82 (57–106)	98.5 (68.8–127.8)	0.131
Month 12	90 (60–111.5)	91 (57.3–123.3)	0.983
Month 24	79.5 (53.3–110)	92.5 (56–118.8)	0.541
Month 36	79 (56.3–99)	118 (51–119.5)	0.457
Month 48	78 (56–87)	52 (47–108.8)	0.513
Last follow-up	80 (54–114)	92 (54.3–119)	0.769
Proteinuria (mg/24 h)			
Baseline	1,672 (1,069–2,988)	1,521.5 (1,163.8–2,895)	0.906
Month 6	820 (190–1,467)	1,153.5 (727–1,474.5)	0.273
Month 12	264 (151–945.5)	931.5 (616–1,286.8)	0.022
Month 24	232.5 (117.8–1,094)	843.5 (490–1,320.5)	0.115
Month 36	357 (111.8–1,287)	840 (314–1,050)	0.804
Month 48	420 (73–874)	403.5 (360–1,017.8)	0.695
Last follow-up	459 (152–1,308)	1,149 (731.3–1,426)	0.092
Follow-up duration (months)	44.6 (23.7–79.5)	32.8 (25.7–62.9)	0.695
Kidney failure, n (%)	3 (13.0)	0 (0.0)	0.536

Notes: *Data are presented as median [interquartile range (25th–75th percentiles)] or number (percentage), as appropriate. C4d positivity was defined as mesangial and/or glomerular capillary deposition. Available sample sizes (C4d-negative/C4d-positive) were 23/10 at baseline and 6 months, 21/10 at 12 months, 20/8 at 24 months, 16/5 at 36 months, and 11/4 at 48 months. All 33 patients had last-follow-up measurements. Between-group comparisons used the Mann–Whitney U test or Fisher's exact test, as appropriate. Analyses were exploratory, with no adjustment for multiple comparisons.* ***Abbreviations:*** *C4d, complement component 4d; eGFR, estimated glomerular filtration rate; S0, absence of segmental glomerulosclerosis according to the Oxford MEST-C classification.*
